# Diagnostic value of real-time PCR of brain mass lesion in HIV-associated toxoplasmic encephalitis: a case series

**DOI:** 10.1186/s13071-020-04443-1

**Published:** 2020-11-10

**Authors:** Bo Liang, Si-Yuan Yang, Jia-Min Chen, Ting-Yu Liang, Hong-Xin Zhao, Xing-Huan Ding, Fang Wang, En-Shan Feng

**Affiliations:** 1 Department of Neurosurgery, Beijing Ditan Hospital, Capital Medical University, Beijing, 100015 China; 2 Laboratory of Infectious Diseases Center, Beijing Ditan Hospital, Capital Medical University, Beijing, 10015 China; 3 Department of Pathology, Beijing Ditan Hospital, Capital Medical University, Beijing, 10015 China; 4 Clinical and Research Center of Infectious Diseases, Beijing Ditan Hospital, Capital Medical University, Beijing, 10015 China

**Keywords:** HIV, Toxoplasmic encephalitis, RT-PCR, Brain mass lesions

## Abstract

**Background:**

Toxoplasmic encephalitis (TE) is a leading cause of brain mass lesions (BML) in human immunodeficiency viruses (HIV)-infected patients. Yet, so far, no accurate diagnostic approach for TE has been developed. Herein, we presented a case series (9 HIV-infected patients with TG confirmed by RT-PCR of BML) to assess the diagnostic value of reverse transcription-polymerase chain reaction (RT-PCR) on TE.

**Methods:**

A total of 9 HIV-infected patients with TE confirmed by RT-PCR of BML were included in this study. Clinical data, including clinical symptoms, blood and CSF analysis, neuroimaging features, histopathological characteristics, treatment, and prognosis, were assessed in all patients. According to the results of RT-PCR of BML, all the patients received oral administration of trimethoprim-sulfamethoxazole combined with antiretroviral therapy (ART). Patients were followed up by telephone or outpatient service.

**Results:**

There were 8 male and 1 female patients; their age ranged from 26 to 56 years-old. The main symptom was intracranial hypertension (6/9). Six patients presented multiple brain lesions, which were mainly located in the supratentorial area (7/9). CD_4_^+^ count ranged from 11 to 159 cells/μl (median 92 cells/μl), and serological HIV viral load 0–989190 copies/ml (median 192836 copies/ml). IgG and IgM against serum TG were positive in 7 and 1 patients, respectively. Moreover, regarding CSF, IgG against TG was positive in 3 patients, while all patients were negative for IgM. The neuroimaging features on MRI showed no specificity. Four patients were diagnosed with TE by histopathological findings. After receiving anti-*Toxoplasma* therapy, 8 (8/9) patients improved clinically to a considerable extent.

**Conclusions:**

The application of RT-PCR of BML, together with conventional methods, may significantly improve the diagnostic efficiency of TE.
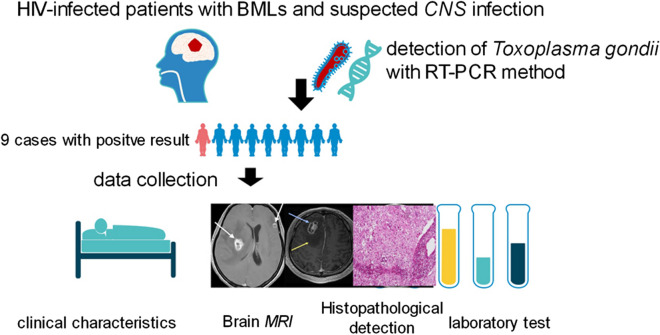

## Background

Toxoplasmic encephalitis (TE) is an opportunistic infection caused by the obligate intracellular protozoan *Toxoplasma gondii* (TG). It usually affects the central nervous system of immunocompromised patients or organ transplant recipients and is a leading cause of brain mass lesions (BML) in HIV-infected patients [1].

The diagnosis of TE is often difficult. Patients with TE usually have no obvious clinical manifestation, while specific abnormal laboratory, neurological imaging, and histopathological findings may be lacking [1, 2]. Even though several diagnostic methods for TE have been developed, the confirmed diagnosis can be merely established through the response to an empiric treatment trial, which may be ineffective or even harmful within 10–14 days. Failure to obtain a timely diagnosis in patients with TE, especially in human immunodeficiency virus (HIV)-infected patients, leads to poor patient outcomes, increased levels of anxiety in patients and families, and a high-cost burden to the health care system [3]. Hence, seeking a useful diagnostic method is of crucial importance.

Over recent years, real-time polymerase chain reaction (RT-PCR), convenient operation, and low risk of laboratory pollution method [4], have been widely used for the clinical diagnosis of infectious diseases of the central nervous system (CNS) [5–8]. For example, Yang et al. [5] used the RT-PCR to detect TG and other pathogens in the cerebrospinal fluid (CSF) of 57 HIV-infected patients with suspected meningitis in central China. Nevertheless, RT-PCR of BML is not currently used for routine tests, especially in HIV-infected patients.

In the present study, we recruited nine HIV-infected patients with TG confirmed by RT-PCR of BML. Clinical characteristics, including clinical symptoms, blood and CSF analysis, neuroimaging features, histopathological characteristics, treatment, and prognosis, were analyzed in all patients to assess the diagnostic value of the RT-PCR-BML method for TE.

## Methods

### Patient recruitment and sample collection

Participants were recruited between May 2013 and April 2020, at the Department of neurosurgery of Beijing Ditan Hospital. Nine participants with RT-PCR-BLM positive for TG were enrolled in this study. Their clinical data, including clinical symptoms, blood examination (included immunoglobulin (Ig) G or M antibodies against TG, CD_4_^+^T lymphocyte count, HIV viral load), CSF analysis (included CSF routine and biochemical tests, CSF HIV viral load, Ig G or M antibodies against TG), neuroimaging features, histopathological characteristics, treatment regime, and prognosis were retrospectively analyzed. The diagnostic criteria of histopathology are a necrotic center, an intermediate zone with an intense inflammatory reaction, and a peripheral zone with an encysted form of TG or with the classical inflammatory response.

According to the results of RT-PCR of BML, all the patients received anti-*Toxoplasma* therapy after the operation, with identical therapeutic regimen: oral administration of trimethoprim-sulfamethoxazole (1440 mg (sulfamethoxazole 1200 mg + trimethoprim 240 mg) Tid), combined antiretroviral therapy (ART), with the regime of highly active antiretroviral therapy (HAART). All patients were followed up by telephone or outpatient service to inquire about survival status; overall survival (OS) was the period from operation to follow-up or death. Brain MRI was reexamined during hospitalization or in outpatient service.

Two trained senior residents collected clinical data. Trained radiologists and pathologists were also invited to assist in making the diagnosis.

During the early stages of admission, BML samples from all patients were collected and stored at -20 °C until further use.

### DNA extraction

For DNA extraction, 500 µg of brain tissue was homogenized by TissueLyser (Qiagen, Hilden, Germany), respectively, in a sterile buffer of PBS with glass beads (4 mm). Nucleic acid was extracted from 200 µl homogenate using a commercial kit (QIAamp Fast DNA Tissue Kit, Cat# 51404; Qiagen), according to the manufacturer’s protocol. Total extracted DNA samples were stored at -80 °C for pathogen’s testing.

### RT-PCR tests

A real-time PCR commercial kit for the detection of TG (Cat# ZD-0075-01) was purchased from Shanghai Liferiver Biological Technology Co. Ltd, China. The TaqMan probe provided in the kit could be used for the amplification and detection for TG in which plasmid DNA and RNAase-free water served as the positive control and negative control, respectively. The 40 μl PCR mix system consisted of 36 μl of RT-PCR reaction mix, 4.0 μl of extracted DNA template from brain tissue, and 0.4 μl of enzyme mixed with the guidance of manufacturer’s description. RT-PCR amplification was performed using SLAN-96P Thermal Cycler (Hongshi Co. Ltd, Shanghai, China), according to the following reaction conditions: 37 °C for 2 min; 94 °C for 2 min; 40 amplification cycles of 93 °C for 15 s and 60 °C for 60 s. Each sample was determined for three replicates. The criterion for a positive result was the cycle threshold (Cq value of ≤ 38 cycles [9].

## Results

### Patients characteristics

Among 9 HIV-infected cases, 8 were males, and 1 was a female. The average age at presentation was 39.78 ± 9.32 years (median age, 39 years; range 26–56 years). The main two symptoms were intracranial hypertension (contained headache, vomiting and visual disturbances) (66.67%, 6/9), and seizures (44.44%, 4/9). Six (66.67%, 6/9) patients presented with multiple brain lesions, which were mainly located in the supratentorial area (77.78%, 7/9) (Table 1).

**Table 1:** Clinical features of 9 patients with RT-PCR of BML positive for TG

Case no./age (years)/gender	Clinical symptoms	Lesion location (S or I)	BML’s number (Sol or Mul)	WBC (blood) (×10^9^ cells/l)	WBC (CSF) (×10^6^ cells/l)	CD_4_^+^ count (cells/µl)	viral load (serum) copies/ml	viral load (CSF) copies/ml	Surgical method (R or B)	IgG/M against TG (serum)	IgG/M against TG (CSF)	PD	OS^b^ (day)
1/42/M	Fever, headache, vomiting	S	Mul	11.37	16	12	106,263	34,687	R	–	–	IL	2235
2/39/M	Headache, dizziness, seizure	S	Mul	2.67	40	33	377,627	181,805	B	IgG +	–	IL	1980
3/26/M	Headache, vomiting, seizure	S	Mul	5.30	5	92	265	na	B	IgG +	IgG +	IL	2461
4/34/F	Headache	S	Mul	5.70	7	59	703,757	na	R	IgG +	IgG +	TE	1318
5/36/M	Seizure	S	Sin	4.43	11	130	51,726	864	R	++^a^	–	TE	191
6/45/M	Hemiplegia	S	Sin	8.46	7	11	919,890	84762	R	–	–	TE	4^c^
7/31/M	Fever, hemiplegia, seizure	S+I	Mul	2.64	6	108	395,491	20	R	IgG +	IgG +	IL	209
8/56/M	Headache, vomiting, Blurry Vision	I	Sin	4.13	20	159	0	na	R	IgG +	–	TE	877
9/49/M	Headache	S	Mul	3.57	48	128	192,836	na	R	IgG +	–	IL	1495

^a^Double positive for both IgG and IgM

^b^The duration from operation to follow-up or death

^c^The only one who is dead

*Abbreviations*: M, male; F, female; S, supratentorial; I, infratentorial; Sin, single; Mul, multiple; WBC, white blood cell; R, resection; B, biopsy; PD, pathological diagnosis; IL: inflammatory lesions; OS, overall survival; na, not available; HIV, human immunodeficiency virus; CSF, cerebrospinal fluid

### Laboratory examination

CD_4_^+^ count ranged from 11 to 159 cells/μl (median 92 cells/μl); HIV viral load in serum ranged between 0–989,190 copies/ml (median 192836 copies/ml) and 20–181,805 copies/ml (median 34,687 copies/ml) in the CSF, with 5 patients available. IgG and IgM against serum TG were positive in 7 and 1 patients, respectively. Moreover, regarding CSF, IgG against TG was positive in 3 patients, while all patients were negative for IgM. The white blood cell (WBC) counts ranged between 2.64 × 10^9^–11.37 × 10^9^/l (median 4.43 × 10^9^/l) in blood and 5 × 10^6^–48 × 10^6^/l (median 11 × 10^6^/l) in the CSF.

### MRI characteristics

**Fig. 1:**
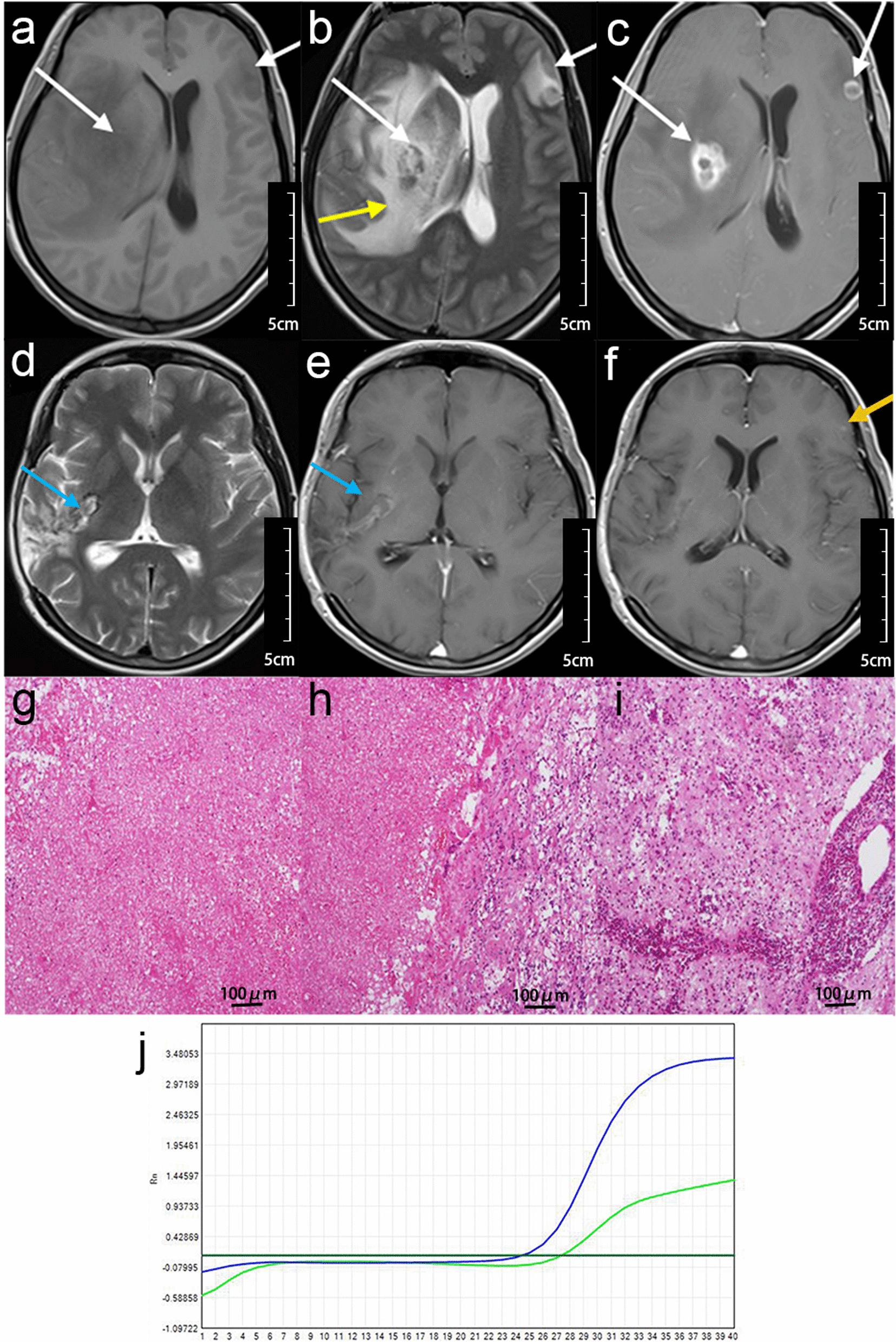
Brain MRI, histopathology and PCR result of case 4. **a**–**c** Images were obtained before the operation. Low signal intensity on T1WI, mixed-signal intensity on T2WI, ring, and nodular contrast enhancement (white arrows), with perifocal edema (yellow arrows). **d**, **e** MRI reexamined nearly 4 weeks after operation and anti-*Toxoplasma* therapy. Residual cavity after operation, perifocal edema reduced significantly, lesions resected completely, no recurrence (blue arrows). **f** Brain lesion at left frontal lobe disappeared (orange arrows). The patient improved clinically and radiographically after 4 weeks of anti-*Toxoplasma* therapy. **g**–**i** Photomicrograph of the histopathological TE, hematoxylin-eosin stained (100×), necrotic center (**g**), intermediate zone (**h**), peripheral zone (**i**) (classical inflammatory response, not contained the encysted form of TG). **j** The RT-PCR of BML revealed TG-positivity

**Fig. 2:**
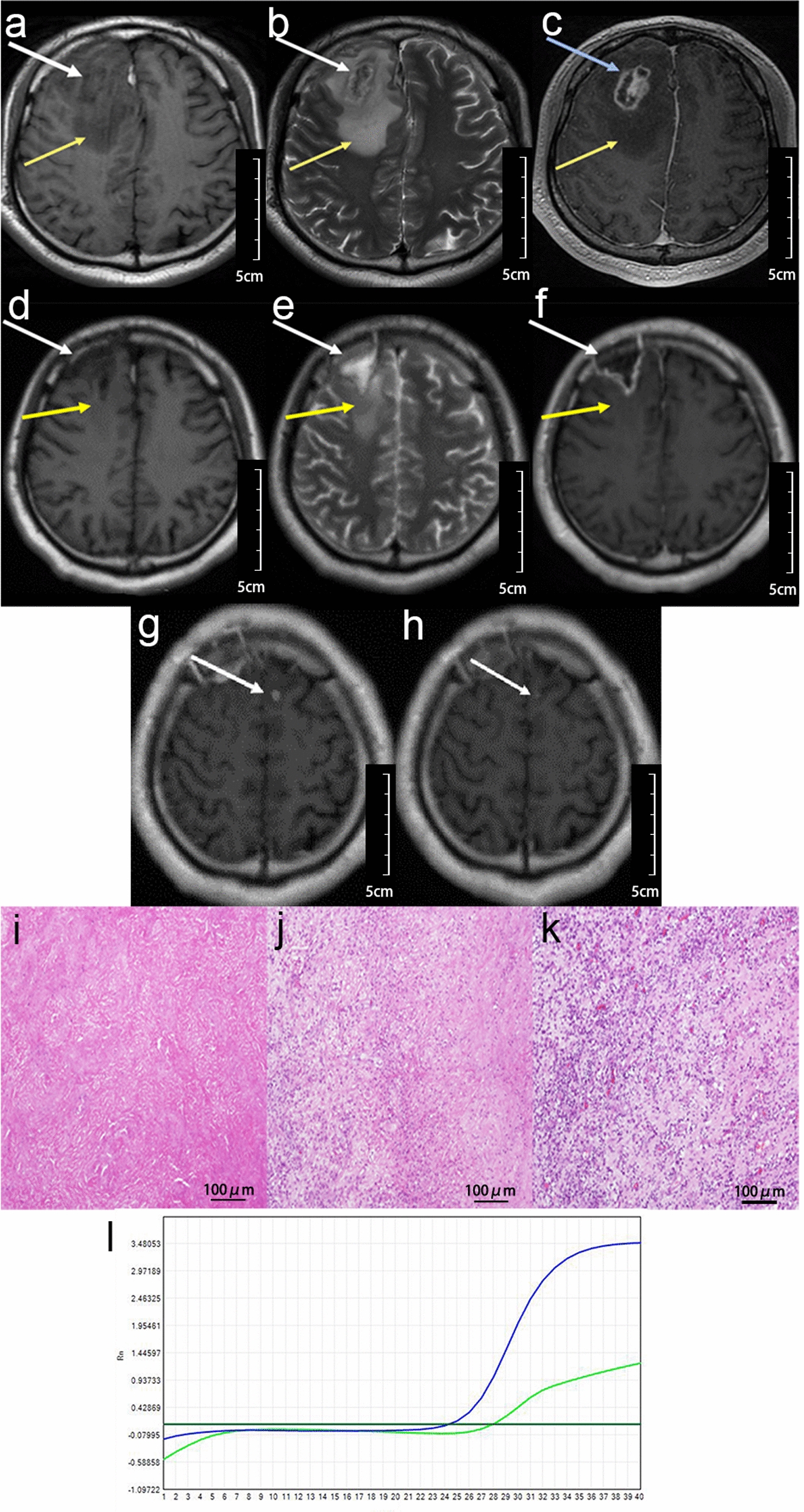
Brain MRI, histopathology and PCR result of case 5. **a**–**c** Images were obtained before operation. Solitary brain mass lesion (white arrows), with perifocal edema (yellow arrows), ring and nodular accumulation patterns of the contrast agent (blue arrow). **d**, **f** Images were obtained 10 days after the operation. Residual cavity after operation (white arrows), perifocal edema reduced significantly (yellow arrows). **g** One new-onset lesion at contralateral side of frontal lobe after the operation 10 days later (white arrow). **h** Images were obtained after 4 weeks of anti-*Toxoplasma* therapy. Brain lesion of new-onset almost disappeared, the patient’s clinical condition obviously improved after 4 weeks of anti-*Toxoplasma* therapy. His OS was 191 days, the seizure never attacked again. **i**–**k** Photomicrograph of the histopathological TE, hematoxylin-eosin stained (100×), necrotic center (**i**), intermediate zone (**j**), peripheral zone (**k**) (not contained the encysted form of TG). **l** The RT-PCR of BML revealed-TG positivity

**Fig. 3:**
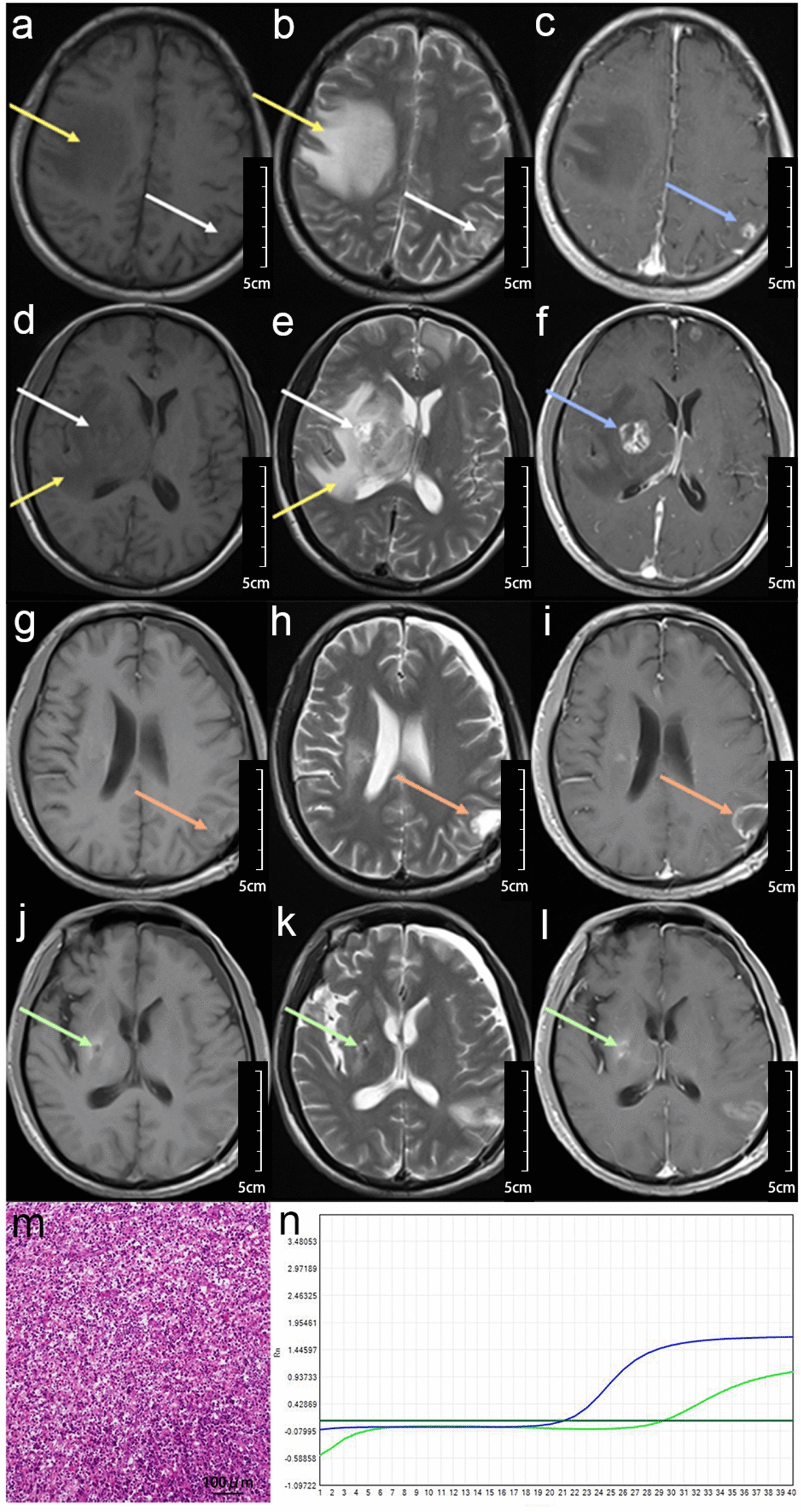
Brain MRI, histopathology and PCR result of case 7. **a**–**f** Multiple brain mass lesion (white arrows), with perifocal edema (yellow arrows), irregular nodular accumulation patterns of the contrast agent (blue arrow). **g**–**l** Images were obtained 3 weeks after anti-*Toxoplasma* therapy. The patient was taken with resection of brain lesion at left parietal lobe (orange arrows). The brain mass lesion at right basal ganglia had been obviously shrunk after anti-*Toxoplasma* therapy (green arrows). **m** Photomicrograph of the histopathological TE, hematoxylin-eosin stained (100×). Non-specific glial cells and microvasculature hyperplasia, infiltration with monocyte and macrophage. **n** The RT-PCR of BML revealed TG-positivity

Brain lesions were located in the basal ganglia, thalamus, cerebellum, and brain stem at the cortical/white matter border, with perifocal edema and occupying effect. These lesions had low signal intensity on T1-weighted images (T1WI), high or mixed signal intensity on T2-weighted images (T2WI), and ring and/or nodular enhancement on contrast, but they all lacked specificity (Figs. 1, 2 and 3).

### Histopathology

Four patients were presumptively diagnosed with TE by histopathology; yet, no *Toxoplasma* cysts were found in these patients. The other 5 patients only presented with non-specific glial cells and microvasculature hyperplasia, infiltration with monocyte, with a diagnosis of inflammatory lesions.

### Treatment and prognosis

Seven patients underwent resection of mass lesions; biopsy was performed in two patients. After receiving anti-*Toxoplasma* therapy combined HAART for 2–4 weeks, clinical conditions improved to a considerable extent in 8 (88.89%, 8/9) patients, and the brain lesions on the MRI obviously shrunk and disappeared. One patient died due to intracranial hemorrhage 3 days after the operation. The OS of the other 8 patients was 191–2461 days (median time 1407 days).

### Typical case description

Participant 4 was a 34-year-old woman who suffered from a headache lasting for a month. She was diagnosed with HIV 20 days before enrollment. Her pre-operative WBC counts in blood were 5.7 × 10^9^/l and 7 × 10^6^/l in CSF. The CD_4_^+^ count was 59 cells/μl. HIV viral load in the serum was 703,757. IgG against TG in serum and CSF was positive, yet IgM was negative. The MRI showed multiple lesions at right basal ganglia and left frontal lobe, with perifocal edema and occupying effect, ring, and nodular contrast enhancement (Fig. 1). Histopathological analysis showed necrotic changes and exudative reaction, and a peripheral zone with an encysted form of TG (Fig. 1).

After the mass lesion was resected (lesion resection at right basal ganglia and decompressive craniectomy), TG was confirmed using an RT-PCR. She then received anti-*Toxoplasma* therapy 3 days later, combined with HAART simultaneously. The reexamination of the MRI was taken nearly four weeks after the operation. No recurrence was observed after treatment. In addition, perifocal edema was significantly reduced, and the brain lesion at the left frontal lobe disappeared. Patient symptoms markedly improved; the OS was 1318 days.

Participant 5 was a 36-year-old man who suffered from seizures lasting for about 20 days. He did not have a history of HIV-infection, so had never received ART before onset of BML. His WBC counts in blood and CSF were 4.43 × 10^9^/l, and 11 × 10^6^/l, respectively. The CD_4_^+^ count was 130 cells/μl. HIV viral load was 51,726 in serum, 864 in the CSF. Serological IgG and IgM were both positive, while those in CSF were negative. The MRI showed a single lesion located at the right frontal lobe, ring, and nodular accumulation patterns of the contrast agent **(**Fig. 2). Histopathological analysis showed necrotic changes and exudative reaction, but no Toxoplasma cysts in the peripheral zone (Fig. 2).

After the mass lesion was resected, TG was confirmed by an RT-PCR. Hence, he received anti-*Toxoplasma* therapy two days later, combined with HAART. Ten days after the operation, the MRI showed one new-onset lesion at the contralateral side of the frontal lobe, but with no symptoms. Four weeks later, the patient was in good condition, and the brain lesion had almost disappeared. His OS was 191 days, and he experienced no recurrence of seizures.

Participant 7 was a 31-year-old man who presented with fever, hemiplegia, and seizure that lasted for 3 months. He also had a history of HIV-infection for about one month. The pre-operative WBC counts in blood were 2.64 × 10^9^/l and 6 × 10^6^/l in the CSF. The CD_4_^+^ count was 108 cells/μl. HIV viral load in the serum was 395,491, 20 in CSF. IgG in serum and CSF was positive, yet IgM was negative. The MRI showed multiple lesions at the right basal ganglia and left parietal lobe, with irregular nodular accumulation patterns of the contrast agent (Fig. 3). Histopathological analysis showed glial cells and microvasculature hyperplasia, infiltration with monocyte and macrophage, with a diagnosis of inflammatory lesions (Fig. 3).

The patient’s mass lesion of the left parietal lobe was resected, and the RT-PCR of BML revealed TG positive, so he was treated with anti-*Toxoplasma* treatment 2 days later, combined with HAART in the meantime. To evaluate the therapeutic efficacy, he underwent an MRI 3 weeks after the anti-*Toxoplasma* therapy, which revealed that the brain lesion at the right basal ganglia had obviously shrunk. Also, his clinical condition significantly improved; the OS was 209 days.

## Discussion

In this study, we investigated the diagnostic value of RT-PCR in BML for detecting TE in HIV-infected patients. The symptoms in nine patients coincided with other CNS infectious diseases ^[7]^. In line with the study performed by Azovtsevai et al. [10], there were more multiple lesions (6/9) in TE, with lower CD_4_^+^ count (below 100 cells/μl), and/or higher HIV viral load (above 50 copies/ml). In accordance with previous research, the IgM in serum and CSF showed low sensitivity [11–13].

**Fig. 4:**
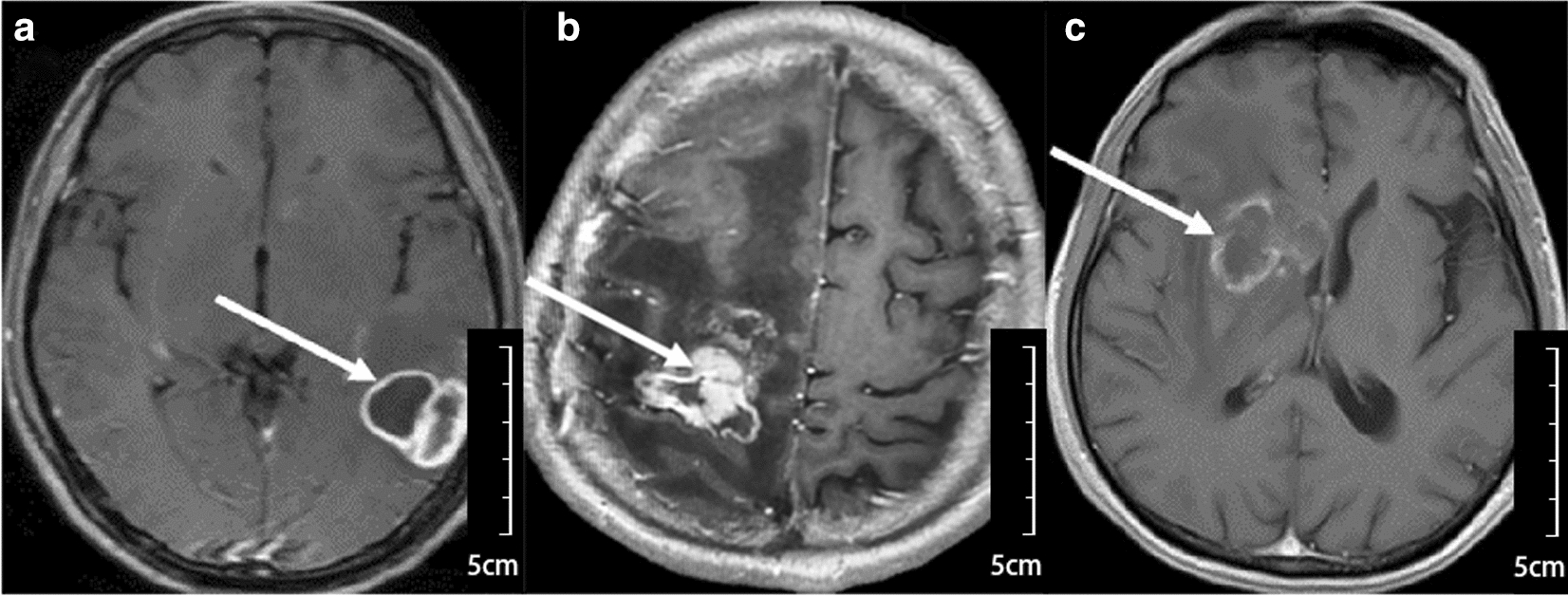
Brain mass lesions in HIV-infected patients by enhanced-MRI examination. **a**-**c** The histopathological diagnosis is lymphoma (**a**) tuberculoma (**b**) TE (**c**), respectively. It is fraught with difficulties to identify from each other by imaging features

The brain MRI is a critical diagnostic method for TE. Dynamic MRI scans are essential for monitoring treatment and outcomes [14–16]. It is generally believed that MRI of TE lacks specificity to distinguish brain mass lesions in HIV-infected patients, especially lymphoma and tuberculoma [1] (Fig. 4). In this study, neuroimaging features on MRI showed no specificity.

RT-PCR is a rapid, comparatively low-cost, and unbiased approach for the molecular diagnosis of diseases. Over recent years, a number of studies have reported on the diagnostic value of RT-PCR in detecting pathogens in CSF [5–8]. Joshua et al. [6] used PCR to identify 17 pathogens in CSF collected from 69 HIV-infected Ugandan adults with meningitis. Among different pathogens, they detected *Cryptococcus* in patients diagnosed with a first episode of cryptococcal meningitis by fungal culture with 100% sensitivity and specificity. They also differentiated between fungal relapse and paradoxical immune reconstitution inflammatory syndrome over recurrent episodes. Similar research was conducted by Radha et al. [7]. They conducted a prospective cohort study of 314 HIV-infected Ugandan adults with suspected meningitis so as to evaluate the etiologies of meningitis by PCR of CSF comprehensively. However, on account of the existence of the blood-brain barrier (BBB), Alfonso et al. [17] and Anselmo et al. [18] showed that CSF-PCR is only sensitive to the diagnosis of meningitis, but not to simple encephalitis. In such instances, RT-PCR of BML may be used as potential alternatives. Moreover, animal models proved that PCR (which can detect 0.1 pg of DNA) is a more sensitive, specific, and rapid approach compared to histopathology when detecting TG in BML [2, 19–21]. Furthermore, RT-PCR of lesion tissue could surveil pathogen loads and disease order of severity using quantitative value analysis.

So far, only a few studies reported on RT-PCR for the detection of TE in BML, especially in HIV-infected patients. In this study, eight of nine patients were diagnosed with TE by analyzing BML using an RT-PCR approach. Consequently, all patients have been treated accordingly (anti-*Toxoplasma* therapy), showing significant clinical improvement. This not only demonstrated that the early treatment of TE is effective [22, 23] but also proved the excellent diagnostic performance and necessity of RT-PCR when detecting TE in BML.

## Conclusions

The application of RT-PCR of BML may significantly improve the diagnostic efficiency of TE. These data lay a foundation for establishing the panel of RT-PCR based on BML to identify a wide range of pathogens, thus further improving the diagnostic efficiency for CNS infectious diseases in HIV-infected patients.

## Data Availability

The datasets supporting the conclusions of this article are included within the article.

## Abbreviations

TE: Toxoplasmic encephalitis
BML: Brain mass lesion
TG: *Toxoplasma gondii*
ART: Anti-retroviral therapy
HIV: Human immunodeficiency virus
PCR: Polymerase chain reaction
CNS: Central nervous system
CSF: Cerebrospinal fluid
Ig: Immunoglobulin
HAART: Highly active antiretroviral therapy
OS: overall survival
WBC: White blood cell
T1WI: T1-weighted images
T2WI: T2-weighted images
BBB: Blood brain barrier
